# First person – Kim Anh Nguyen

**DOI:** 10.1242/bio.048769

**Published:** 2019-11-29

## Abstract

First Person is a series of interviews with the first authors of a selection of papers published in Biology Open, helping early-career researchers promote themselves alongside their papers. Kim Anh Nguyen is first author on ‘Optimization of high endoglucanase yields production from polypore fungus, Microporus xanthopus strain KA038 under solid-state fermentation using green tea waste’, published in BIO. Kim Anh conducted the research described in this article while a master's student in Saisamorn Lumyong's lab at the Center of Excellence in Microbial Diversity and Sustainable Utilization, Chiang Mai University, Thailand. She is now a PhD student in the lab of Otakar Strunecky at the Faculty of Fisheries and Protection of Waters, The University of South Bohemia, Czech Republic, investigating microbial biotechnology.


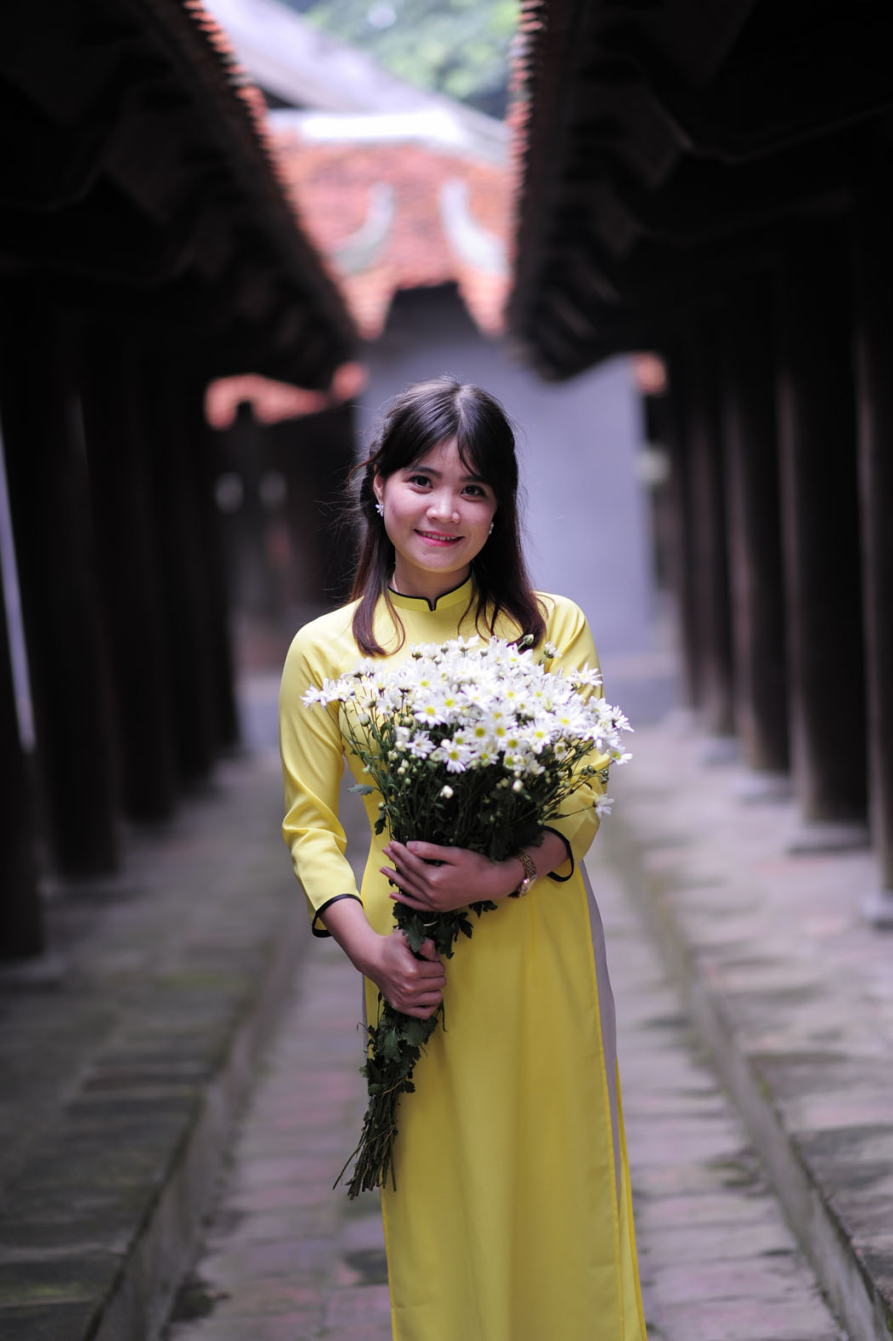


**Kim Anh Nguyen**

**What is your scientific background and the general focus of your lab?**

After finished my bachelor's degree from Hanoi University of Natural Resources and Environment, I continued to be a master's student for almost 3 years at Chiang Mai University, in Thailand. I also worked as a research assistant to have more of a chance to research microorganisms, such as fungi, bacteria, microalgae and virus. Subsequently, I decided to choose fungi to be the strain to work on with enzyme production. My work then focused on producing a high yield of enzymes for industrial purposes.

**Figure:**
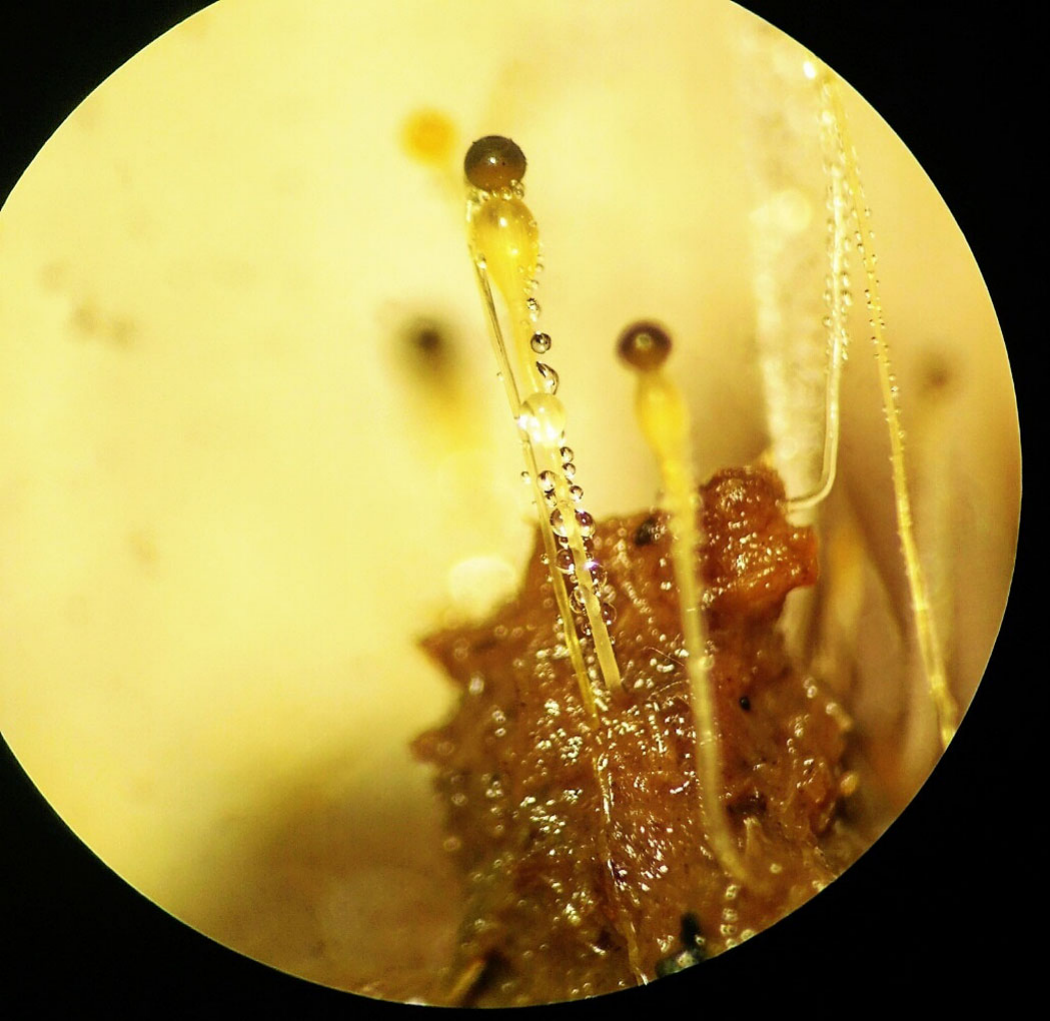
**Collecting a sample in the field.**

**How would you explain the main findings of your paper to non-scientific family and friends?**

Enzymes increase the rate of a chemical reaction without themselves being changed by the reaction. Nowadays, enzymes are used in almost all industrial fields. However, it is very expensive to get the pure enzyme. To reduce the cost of enzyme production, we utilized the waste of tea agriculture after fermentation and optimized the conditions and we determined the optimal conditions for enzyme production at a low cost.

**What are the potential implications of these results for your field of research?**

This research demonstrated that *Microporus xanthopus* was a remarkable species of polypore fungi in producing endoglucanase activity. The potential of enzyme production using *M. xanthopus* is that it can be used to replace other microorganisms without decreasing the high yields.

**What has surprised you the most while conducting your research?**

At the beginning of this project, I conducted many tests, but the results from those tests were failures. It got me down and it took a while to reset my experiments. The change of enzyme volume was very a sensitive procedure, it can change fast and significantly when the conditions for fermentation are changed. Some experiments surprised me because the results changed extremely quickly.

“Without publication, science is dead.”

**What changes do you think could improve the professional lives of early-career scientists?**

Without publication, science is dead. However, to get peer reviewed and into a high-quality publication is not easy. It asks the authors to collect a lot of information and to do a lot of research. And where can we get believable information? From you and also me. Why don't we share our findings in our publication, and when we give, we receive.

**What's next for you?**

I will be a PhD student soon, I will have more chances to continue to work in microorganisms, and also I can find more interesting and useful things in life.
